# Intracerebral Bleeding and Massive Pericardial Effusion as Presenting Symptoms of Myxedema Crisis

**DOI:** 10.1155/2017/8512147

**Published:** 2017-02-01

**Authors:** M. Kirsch, C. Rimpau, C. H. Nickel, P. Baier

**Affiliations:** ^1^Clinic of Internal Medicine, University Hospital of Basel, Basel, Switzerland; ^2^Department of Anesthesiology and Intensive Care, University Hospital of Basel, Basel, Switzerland; ^3^Department of Accident and Emergency, University Hospital of Basel, Basel, Switzerland

## Abstract

The endocrinological emergency of a fully blown myxedema crisis can present as a multicolored clinical picture. This can obscure the underlying pathology and easily lead to mistakes in clinical diagnosis, work-up, and treatment. We present a case of an unconscious 39-year-old patient with a medical history of weakness, lethargy, and findings of hyponatremia, intracerebral bleeding, and massive pericardial effusion. Finally, myxedema crisis was diagnosed as underlying cause. Replacement therapy of thyroid hormone and conservative management of the intracerebral bleeding resulted in patient's survival without significant neurological impairment. However, diagnostic pericardiocentesis resulted in life-threatening pericardial tamponade. It is of tremendous importance to diagnose myxoedema crisis early to avoid adverse health outcomes.

## 1. Introduction

Myxoedema crisis is an almost forgotten cause of multiorgan failure with a mortality of about 25–50% [1, 2]. Nonspecific presenting symptoms such as lethargy and weakness confront the clinician always with a broad spectrum of differential diagnoses and may obscure the underlying disease [3–5].

## 2. Case Presentation

A comatose, circulatory stable, but hypoventilating patient was intubated at the scene and brought in to the emergency department by ambulance. The past medical history, which was provided by his grandmother, revealed that this 39-year-old man had lived with her for many years. The previous weeks prior to presentation he complained of fatigue and recurrent nose bleeds, and he was eating irregularly. At the age of 3 he had been operated on a benign cerebral gangliocytoma and had been on antiepileptic medication because of generalized seizures for some years. The patient had not seen a doctor for many years. Physical examination revealed a patient in a dishevelled condition with bilateral vesicular breathing and distant but regular heart sounds without any murmur, rare bowel sounds, no tenderness to deep palpation, and nonpitting oedema of the legs. His body temperature was slightly elevated (38.5°C). The neurologic examination showed equal pupils reactive to light symmetric muscle tone and absence of plantar reflexes.

On admission, laboratory data showed significantly decreased levels of sodium and potassium (sodium: 120 mmol/L, potassium: 2.9 mmol/L) as well as increased levels of creatine kinase (overall-CK: 967 U/L, CK MB 6.7 *µ*g/L (norm CK MB < 7.7 g/L in males)) and glutamate- oxalacetate-transaminase (AST: 62 U/L). The coagulation panel demonstrated a slightly increased international normalised ratio (INR: 1.4) and partial prothrombin time (PTT: 37 s). The complete blood count showed a normochromic normocytic anaemia (haemoglobin: 114 g/L), with normal platelets and leucocytes. The arterial blood gas on admission showed an alkalosis (pH 7.43) with an increased bicarbonate of 31.8 mmol/L (base excess of +7), but as well increased pCO_2_ of 6.4 kPa. The remaining results were unremarkable including normal liver function tests, glucose levels, and creatinine and urea. A urinary toxicological screening showed no signs of intoxication with benzodiazepines, opiates, amphetamines, tricyclic antidepressants, acetaminophen, or salicylate.

**Figure 1:**
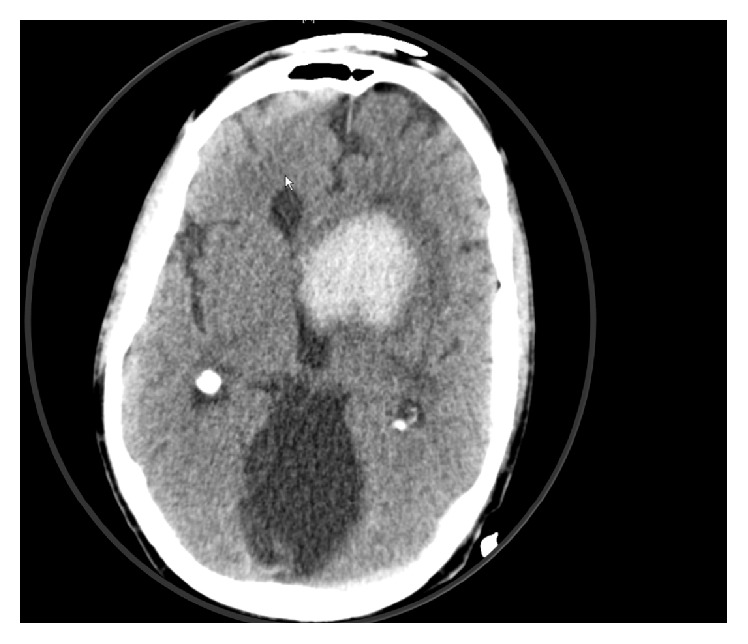
Angio-Head-CT shows a frontobasal left sided intracerebral bleeding with irruption into the ventricles and an enlarged ventricle system.

**Figure 2:**
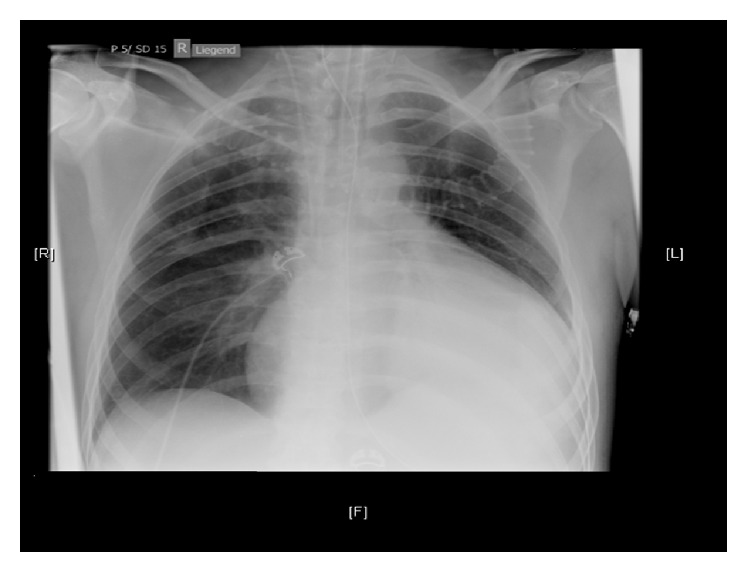
Chest radiograph (anteroposterior view) shows no pulmonary infiltrates or effusions, no pneumothorax, and no cardiac congestion but an enlarged heart with a water-bottle configuration.

**Figure 3:**
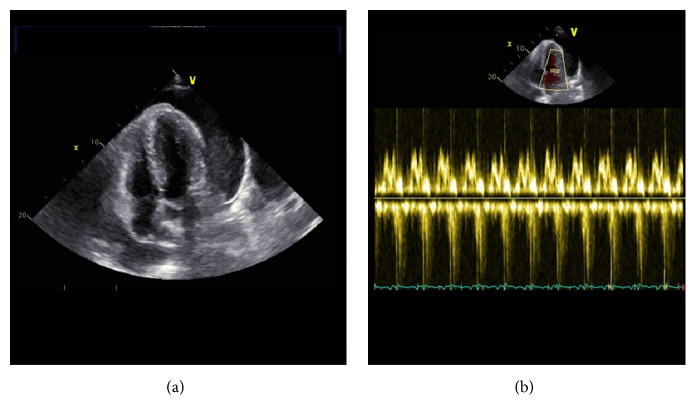
Echocardiogram shows significant pericardial effusion (a) without significant impact on right ventricular function proved by normal mitral inflow (b).

The electrocardiogram (ECG) showed a sinus rhythm (85 bpm), normal axis, and an incomplete right bundle branch block (RBBB). The QTc time was within normal range. A computer tomography with angiography of the cerebral arteries (Angio-CT) showed a frontobasal intracerebral bleeding with irruption into the ventricles. A cerebral aneurysm and skull fracture were excluded (Figure 1). A chest X-ray (CXR) showed impressive cardiomegaly without any signs of chest infection or congestive heart failure (Figure 2). An echocardiogram showed a large pericardial effusion with swinging heart but without compromise of right ventricular function and a normal variation of mitral inflow (Figure 3). Significant valve dysfunction was excluded and normal systolic and diastolic left ventricular function and size of heart chambers confirmed. The patient was transferred to the intensive care unit (ICU). Analgosedation with propofol and fentanyl and Continuous Positive Airway Pressure (CPAP) ventilation was continued (positive end expiratory pressure (PEEP): 5 and pressure support (ASB): 5–10 mmHg, inspiratory oxygen concentration FiO_2_: 40%). To maintain a MEAN blood pressure of 65 mmHg low-dose support with noradrenaline was needed. A supplementation of potassium and sodium was performed. An electric encephalogram (EEG) showed a left frontopolar epileptic focus, so that therapy with levetiracetam was started. Further, diagnostic needle pericardiocentesis was performed and 1300 mL of slightly haemorrhagic pericardial fluid evacuated (glucose 11.5 mmol/L, protein 73 g/L, albumin 44 g/L, lactate dehydrogenase (LDH) 173 U/L, red blood cells: few, and white blood cells 0,21 × 10^9^ (25% neutrophils, 2% lymphocytes)), Gram stain and bacterial cultures were negative, cytology was negative for malignant cells, and polymerase chain reaction (PCR) for tuberculosis was negative.

About four hours after this procedure the patient went into cardiac arrest (pulseless electrical activity, PEA) caused by cardiac tamponade. Though the pericardial tube was still in place it was obstructed by coagulated blood. A second needle pericardiocentesis was performed and a significant amount of deeply red haemorrhagic cardiac effusion could be removed, which resolved the patient's deterioration immediately. Further work-up of the hyponatremia (120 mmol/L) could exclude pseudohyponatremia (serum osmolality: 267 (normal range: 280–300 mmol/kg)) and adrenal insufficiency by an adrenocorticotropin hormone (ACTH) stimulation test (cortisol basal: 919 nmol/L, 30 min after stimulation with 250 *μ*g ACTH: 1137 nmol/L, and 60 min: 1235 nml/L). Urine osmolality was 610 mmol/kg and urine sodium content was 39 mmol/kg. Additionally, the thyrotropin level was significantly increased at 112 (normal range 0.33–4.49 mlU/L) accompanying T4 and T3 levels of 1.2 (normal range: 66–181 pmol/L) and 0.4 (normal range: 1.1–3.2 pmol/L), respectively. Thyroid peroxidase (TPO) antibodies could be shown to be significantly increased to 294 IU/mL (normal range: 100–200 U/L). As Hashimoto thyroiditis was found as underlying pathomechanism, the patient was treated with 100 *μ*g levothyroxine once daily and the patient could be extubated five days after admission. Fortunately he was only slightly neurologically impaired (GCS 14, with no signs of cerebral nerve paralysis but decreased muscular strength in all four extremities (M3/4)). Due to recurrence of the pericardial effusion in echocardiographic follow-up, a thoracoscopic pericardial fenestration was performed about 3 months after the ICU stay.

## 3. Discussion

We describe a case of a 39-year-old comatose patient presenting with the major findings of a spontaneous intracerebral bleeding, electrolyte disturbance (low sodium and potassium), and a massive pericardial effusion. Adding the more subtle findings of fatigue and recurrent nose bleeding over the last weeks, a nonpitting oedema on the lower legs, an elevated bicarbonate as a sign of a chronic hypoventilation, an elevated creatine kinase (CK-MM), and a slightly altered coagulation panel, this patient showed several signs and symptoms of hypothyroidism. The fact that the patient was intubated at the scene, the rare presentation of myxedema crisis in our days, and the missing cardinal symptoms of bradycardia and hypothermia might have obscured the clinical picture of a fully blown myxedema crisis which led to delayed diagnosis.

The association of hypothyroidism with minor bleeding events (gum bleeding, bruises, and menorrhagia) is widely known; however only a few reports of hypothyroidism as risk factor for intracerebral bleeding and its effect on morbidity and mortality are published to date [6]. The initial coagulation panel of our patient showed a slightly increased PTT and INR, which resolved spontaneously. But it should be noted that some authors suggest acquired von Willebrand's (vW) syndrome as the main underlying factor of impaired coagulation in patients with hypothyreosis [7]. vW syndrome does not result in significant alteration of routine coagulation panel although the patient's coagulation could be impaired significantly. The molecular mechanisms of interaction between thyroid hormones, thyroid receptors, and vW-factor have been suggested to be secondary to a reduced synthesis and/or secretion of vW-factor by vascular endothelial cells. This hypothesis was built on in vitro experiments with human umbilical vein endothelial cells (HUVECs), where fT3 exposure resulted in increased mRNA expression and protein synthesis of vW-factor [8]. Though these data could not be confirmed by Diekman et al. [9] it could be shown that initial treatment with desmopressin (DDAVP, 0.3 *μ*g/kg) accompanied with thyroid hormone replacement significantly enhances platelet adhesiveness and plasma concentrations of factor VIII and vW-factor in ten patients with hypothyroidism [10]. In our case DDAVP treatment seemed not to be advisable because it might have potentiated the tendency of hyponatremia. However, treatment with fresh frozen plasma (FFP) or recombinant vW-factor and early thyroid hormone replacement could be a promising treatment of patients with hypothyroidism in cases of significant bleeding or inevitable interventional procedures.

Without the ECG findings of low voltage or QRS alternans and a hemodynamical stable patient we have been surprised by the finding of a significant pericardial effusion on chest X-ray (CXR). The decision to proceed to an immediate needle pericardiocentesis was largely driven by the suspicion of a hematologic disease, autoimmune disease, or an underlying malignancy, which could explain both the massive cardial effusion and the intracerebral bleeding. Lymphoid malignancies, collagenoses, and infections have been reported to be common causes for pericardial effusions [11]. The analysis of the patient's pericardial fluid yielded a benign noninfectious pericardial effusion but resulted in a cardiac arrest caused by cardiac tamponade.

Pericardial effusion and even cardiac tamponade as presenting symptoms of hypothyroidism are rare but have been described earlier [12–16]. In the general population, the risk of major complications after pericardiocentesis is reported to be about 5% [17]. Although the major adverse event of cardiac tamponade after pericardiocentesis could have been caused by cardiac puncture or laceration, it has to be pointed out that the risk of needle pericardiocentesis in patients with hypothyroidism could be far greater than in the general population. The effects of thyroid hormones on the cardiovascular system have been reviewed in detail by Klein and Ojamaa. It has to be noted that the systemic vascular resistance is markedly increased whereas cardiac contractility is decreased in hypothyroidism. Although the blood pressure might not be significantly affected as a result of opposing effects, the cardiac work might be significantly increased because of the elevated cardiac afterload [18]. Additionally, it was reported that the cardiovascular system is significantly depressed as the response to catecholamines might be significantly reduced by downregulated expression of adrenergic receptors in hypothyroidism [19]. Lim et al. described a pericardial decompression syndrome, which resulted in severe hemodynamic instability after diagnostic needle pericardiocentesis in a patient with hypothyroidism [16]. Therefore, the body cannot counteract the hemodynamic effects of a cardiac tamponade with tachycardia and peripheral vasoconstriction [16]. Finally it has to be questioned whether a pericardiocentesis is needed at all because complete resolution of pericardial effusion after starting replacement therapy of thyroid hormone has been reported [15].

Finally, the work-up of hyponatremia resulted in the diagnosis of myxedema crisis caused by severe autoimmune hypothyroidism. Though questioned by some reports [20, 21] the correlation of hyponatremia and hypothyroidism is widely accepted. The pathophysiologic mechanisms of hypothyroidism-associated hyponatremia are believed to be the inability of the body to excrete free water caused by a combination of decreased glomerular filtration rate and accumulation of interstitial mucopolysaccharides with fluid retention, which results in decreased effective arterial blood volume and relatively high levels of antidiuretic hormone (ADH) because of decreased cardiac output and stimulation of the carotid sinus [22]. However, our patient had as well an intracerebral bleeding, which could result in a syndrome of inappropriate antidiuresis (SIAD) or cerebral salt wasting syndrome. The inordinately high urinary sodium excretion and urinary osmolality in combination with a normal serum level of creatinine and urea as well as normal urine output suggest that SIAD might play a minor additional role.

The final diagnosis and treatment of myxedema crisis resulted in significant improvement of the patient. He could be extubated and discharged with only minor neurological impairment.

The chosen route and dose of supplementation of levothyroxine were not in line with international guidelines [23, 24]. As was demonstrated by the work of Ladenson and Ridgway the treatment with 50 *µ*g L-triiodothyronine or 100 *µ*g L-thyroxine intravenous was able to result in reversal of blunted ventilator responsiveness, increase in cardiac inotropy, renal excretion of water load, and basal metabolic rate within hours to days after the start of treatment [25, 26]. Therefore, despite a significant improvement of the patient over the first days with our supplementation of 100 *µ*g L-thyroxine per os, starting with a loading dose of 500 *µ*g L-thyroxin, choosing the intravenous route, or even adding L-triiodothyronine and steroids as recommended by international guidelines might have decreased the days of artificial ventilation and ICU stay.

## 4. Conclusion

Although psychiatric disorders or substance abuse is main reasons for weakness, lethargy, and fatigue in young patients [3–5], it is of tremendous importance to diagnose endocrinologic disorders as myxedema coma early to start causative treatment. Invasive diagnostic procedures if not deemed life-saving should be postponed because of a higher risk of complications caused by impaired coagulation and hypometabolic state [8, 15, 16].
